# Shared care involving cancer specialists and primary care providers – What do cancer survivors want?

**DOI:** 10.1111/hex.12551

**Published:** 2017-05-03

**Authors:** Sharon Lawn, Julia Fallon‐Ferguson, Bogda Koczwara

**Affiliations:** ^1^ Flinders Human Behaviour & Health Research Unit Department of Psychiatry Margaret Tobin Centre Flinders University Adelaide SA Australia; ^2^ Former National Manager of PC4 Primary Care Collaborative Cancer Clinical Trials Group The University of Western Australia Perth WA Australia; ^3^ Department of Medical Oncology Flinders Medical Centre Flinders Centre for Innovation in Cancer Flinders University Bedford Park SA Australia

**Keywords:** cancer survivors, chronic condition management, primary care, shared care, shared decision making

## Abstract

**Background:**

Cancer survivors are living longer, prompting greater focus on managing cancer as a chronic condition. Shared care between primary care providers (PCPs) and cancer specialists, involving explicit partnership in how care is communicated, could ensure effective transitions between services. However, little is known about cancer patients' and survivors' preferences regarding shared care.

**Objective:**

To explore Australian cancer survivors' views on shared care: what cancer survivors need from shared care; enablers and barriers to advancing shared care; and what successful shared care looks like.

**Setting and Participants:**

Community forum held in Adelaide, Australia, in 2015 with 21 participants: 11 cancer survivors, 2 family caregivers, and 8 clinicians and researchers (members of PC4‐Primary Care Collaborative Cancer Clinical Trials Group).

**Intervention:**

Qualitative data from group discussion of the objectives.

**Results:**

Participants stressed that successful shared care required patients being at the centre, ensuring accurate communication, ownership, and access to their medical records. PCPs were perceived to lack skills and confidence to lead complex cancer care. Patients expressed burden in being responsible for navigating information sharing and communication processes between health professionals and services. Effective shared care should include: shared electronic health records, key individuals as care coordinators; case conferences; shared decision making; preparing patients for self‐management; building general practitioners' skills; and measuring outcomes.

**Discussion and Conclusions:**

There was clear support for shared care but a lack of good examples to help guide it for this population. Recognizing cancer as a chronic condition requires a shift in how care is provided to these patients.

## 1 INTRODUCTION

Improvements in screening, early detection, and treatment of many cancers have meant survival rates have improved significantly.^1^ Cancer survivors are living longer and are a growing population within the community.^2^, ^3^ In addition to any pre‐existing co‐morbidities, they are also more likely to develop chronic conditions as a consequence of their longer survival, and as an adverse consequence of cancer treatments.^4^ Hence, there has been a growing focus on models of care that focus on management of cancer as a chronic condition, which promote early re‐engagement of the primary care provider (PCP) to support transition from the acute oncology sector back to primary care and include stratification of risk of recurrence, promotion of self‐management for low‐risk patients, and mechanisms for rapid access back into the specialist setting. These new models require acceptance by health‐care providers and cancer survivors that PCPs can play an important role in delivery of many aspects of cancer care.^5^ However, this is frequently hindered by fears about PCPs' lack of cancer‐specific expertise and access to specialist advice when needed.^6^, ^7^ Much less is known about patients' and survivors' preferences regarding shared care where roles and responsibilities are more clearly defined.

Shared care as defined by the American Society of Clinical Oncology (ASCO) is, “the communication process between the oncology specialist and primary care provider and successful transition of the patient from the oncology setting to primary care setting post treatment”. It is a partnership of PCPs and cancer specialists using an explicit format with agreed processes and deliverables.^8^, ^9^ It is distinct from hospital‐led care, primary care‐led care, or a setting where multiple providers deliver care to the same patient without overtly working together. A key enabler of effective shared care is the availability of clinical information across the care continuum.^10^ In this context of shared care, both the specialist and the PCP maintain on‐going involvement in patient care and in doing so, share information and clinical responsibilities and agree on common processes.

We explored Australian cancer survivors' views on shared care between cancer specialists and PCPs as part of the development and consultation of the PC4 *Principles Statement on Shared Care in Cancer*. PC4 is the Australian Primary Care Collaborative Clinical Trials Group, a multidisciplinary clinical trials group aimed at fostering research in primary care and cancer. The consultation process included two key forums: one with PCPs already delivering shared care successfully in areas other than cancer (for example, obstetrics and diabetes) and other a community forum with cancer survivors and carers representing diverse cancer advocacy and support organizations. The latter was a three‐hour face‐to‐face meeting to discuss various aspects of shared care in cancer. The objectives of the discussion were to provide feedback on shared care around the following four broad question areas:

- What do cancer survivors need from shared care?
- What can be done to advance shared care?
- What are some of the barriers to overcome?
- What would successful shared care look like?

## 2 METHODS

### 2.1 Participants and recruitment

The community forum included 21 participants, 14 of which were women: 11 cancer survivors, 2 family carers, and 8 members of PC4 (clinicians and researchers). It was aligned with the 2015 Survivorship Conference held in Adelaide, Australia. This conference attracts cancer survivors and family (and researchers and clinicians) from across Australia and internationally. Participants were recruited via email communication sent by PC4 to its database of stakeholders located across Australia (including cancer patients and survivors, family of cancer survivors, clinicians, and academics), and by Cancer Voices Australia (a major Australian cancer survivor advocacy group) to their members. The forum was held the day before commencement of the conference. Participation was voluntary.

In my experience, you go to your GP [general practitioner] with an ache or a lump or a something, whatever it is. They send you off for a biopsy and blood tests. As soon as the results come back, they ship you off like a shot from a cannon as quickly as they can to whichever specialist chain they are in, then nothing is really shared after that.

### 2.2 Data collection

The forum facilitator was Professor Bogda Koczwara, conference convenor, national researcher, and advocate in survivorship care. The forum discussion was audio‐recorded and professionally transcribed to ensure accuracy of recall and to enhance data analysis by capturing all comments during the discussions.

### 2.3 Data analysis

A full transcript of the forum discussion was provided to all participants for their feedback and confirmation of its content approximately 1 week after the forum. Following this, the two researchers independently undertook interpretive content analysis of the data, informed by the framework of the broad questions that guided the forum discussions. Interpretive content analysis was chosen to analyse the data, given the focus on clear domains of interest and the single forum structure for data collection.^11^ Analysis involved reading and re‐reading the transcript, organizing responses under each question area, followed by descriptive open coding representing interesting features of the data. The researchers then met to discuss the coding process, determine any differences in perspective and interpretation of meaning, reach consensus, and finalize the draft analysis prior to seeking electronic feedback from forum attendees on this agreed draft. Upon receiving this feedback, the researchers then undertook further interpretation and shared discussion, grouping coded ideas into final subthemes before producing a report using pertinent statements using participants' own words to accurately and succinctly reflect the ideas arising from this process.

### 2.4 Ethical considerations

The project received ethics approval from the Flinders University Social and Behaviour Research Ethics Committee (No.6802). All participants received a Participant Information Sheet and provided their signed consent to participate and to have their views recorded as part of the forum. Participants could withdraw their consent by approaching the primary researcher privately at the conclusion of the workshop, or by contacting them within 1 week of its occurrence.

## 3 RESULTS

Results are reported across three main themes that were informed from the framework of questions used to facilitate forum discussions. Direct quotes from cancer survivor participants exemplify the themes, with clinicians and researchers contributing to the discussion of ideas on which themes are based.

### 3.1 What cancer survivors need from shared care

There was consensus from forum attendees that successful shared care required the patient being at the centre of the shared care arrangement, and they provided a range of ideas to explain what this actually meant from a conceptual and practical standpoint.

Survivor participants described a range of reasons for their need to be at the centre of the process; the first of these related to ensuring accuracy of communication to ensure mistakes in care were not made by health professionals working within a fragmented communication system:

A knowledgeable patient is the ultimate safeguard. If I didn't know what was going on, mistakes would have happened, which would have been bad….

My GP has used me as his main source of information because the emails just don't seem to get through to him.

I've needed to keep my own records. There are no such things as care plans. There should be and I'm advocating very strongly for them. I have a five‐page spread‐sheet which I've developed which I take with me because no‐one else has all of that information. I've moved through different hospitals, through different doctors so I've needed to manage…

There was discussion of the tension between being “passed around” by various health‐care providers vs self‐managing a navigation role, which requires effort and skill to do so.

I learned that I had to ask the questions and that took time to discover that … it didn't actually come naturally so I had to play an active role in that…because when you're new to that whole system, you don't know you've got rights. You don't know that there are other possibilities so I think being made aware of that … it starts to give patients some power.

They noted that patients' capacity to take on this role might vary, as might the dynamics between the individuals involved in the shared care process, including expectations of who does what, who has expertise, and who is trusted to perform particular roles. There was a perception that GPs lack skills and confidence to lead the management of the complexities of cancer care.

GPs in my experience are often very scared of complex cancer patients. They don't have the training; they don't know what to do. You go in with the slightest little ache and pain and you're shot off for scans and x‐rays and what have you that may or may not be necessary but they just don't know what to do.

As a consequence of problems including a lack of leadership and navigational support from health professionals across the system, participants discussed the burden of being responsible for navigating the information sharing and communication process between health professionals and services. They agreed that, although this was “daunting at times” and could be “scary,” there wasn't anybody who was better at knowing the patients' needs than the patients themselves. The lesson that the group said they took from this was that patients' involvement and burden was an inevitable part of the process and therefore it was important to make the partnership process with patients easier.

It's about being equal in it … about being understood as being a vital cog in it and I think that's part of it. You probably will never stop people having their own approach to it but if you actually were all working in the same sort of paradigm of it, particularly with the types of technology and records that can be done now in that environment, it would help.

An important feature of this “inevitable” role for patients was their need for greater transparency and timeliness of communication with and between health professionals. Central to this was greater ownership and access to their medical records, given the reality of their central role as navigators of the care process.

It's being in charge of your own records – you want them but you have to beg and scrape to even get hold of them. Even blood test results…Sometimes I can get a copy, sometimes I can't. I go back to my GP and she says, “Oh if you can get hold of your results to bring to me that would be fantastic.”

### 3.2 Advancing shared care

Forum participants were then asked about what they thought would help to address their needs and improve shared care. The use of shared electronic health records was overwhelmingly supported as a potential solution to improve shared care for cancer survivors because it was perceived to improve ownership and access to information by all concerned (including the patient), and therefore to minimize fragmentation, errors, and miscommunication across systems of care.

To look after your own health and self you need to have an overall picture of where you are at across all of your specialists, across all of your treatments, hospitals, your future, your past. It's got to be managed and kept together and I think the only way to do that is to allow patient input and allow patient access.

That whole issue of patient data and sharing patient's results needs to be sorted out before we're going to be able to have meaningful shared care that we can all be part of this same process.

However, participants noted the need for such an approach to receive more political support to ensure its successful implementation into broader practice. They saw patients as important advocates for shared electronic health records.

one of the problems … was that it originally was set up as an opt‐in model and there was very low uptake, not surprisingly, because there wasn't a huge push really… A shift in the legislation to make it an opt out model which might potentially increase the actual use of the record eventually; but there needs to be strong political commitment to make it happen as well. I think that's what's lacking at the moment.

Sharing expertise and responsibility was also emphasized as a means to advance shared care. A clearer assessment of patients' holistic needs from the outset was perceived as one way that health professionals might see beyond their own role and recognize that other health professional roles might be needed within a shared care approach.

If a health care provider were to look at those needs, they may say “I am very capable of meeting those needs” or they may say “I'm capable of meeting the needs from one to number five, but not needs six and nine because I don't have the skills or resources or a bit of both”. So, the moment you identify that some needs cannot be met just by you, then you're almost forced to face the possibility of sharing care with somebody else (Health Professional).

When asked why they thought routinely shared care plans do not currently happen, participants discussed issues with duplication and organizational inertia, with the latter hampered by varied privacy legislation across each jurisdiction and concerns about confidentiality and information sharing. A proposed solution was lobbying for government to show leadership at the federal policy level to create more consistent legislation.

I know it doesn't happen now because I've got a 20‐year journey to tell me that it doesn't. I know that all of the different hospitals and all the different departments within the different hospitals are working on care plan models. My concern is that what we're going to have is 100 different care plans that don't talk to each other and what I would like to see is one model that everybody can link into. I don't see why it has to be so.

### 3.3 What good shared care looks like

Following the detailed discussion of barriers and enablers to successful shared care, participants offered a range of suggestions for what they thought effective shared care looked like. These are discussed in the following sub‐themes.

#### 3.3.1 Key individuals as Case Managers/Care Coordinators

Participants discussed the idea of a clear key contact person or care coordinator/case manager to bridge the hospital/primary care sectors and to be a navigation point and for problem‐solving any communication issues.

I've got a perfect example. I had a neurosurgical nurse who said, “Here's my card, call me if you have a problem,” so I had a one stop contact.

Set up care coordinators so your GPs know who to speak to, “I have a cancer patient, I can ring the hospital; this is the person I call or email”

Although participants recognized that such an approach might create more problems with fragmentation, one circumstance where this was seen as particularly important was where the patient had multiple health conditions.

It is different if someone's got diabetes as well as cancer so that case manager actually having the big picture – not that they're the go to person for everything – it's just that they do have their eyes on the picture.

Akin to the above dialogue about patients as navigators, participants suggested that case managers could also be individuals who were not health professionals.

I've recently been a case manager for a friend that I met through a chat room … with a depressive illness and several other medical situations. She had more specialists than I could name, a bigger medical history than I could take down in two days but I worked with her one on one over a matter of weeks to pull together a medical history that could go to her anaesthetist that could go to her surgeon. I worked with her on admission forms for that hospital, for that hospital and for that hospital. I was her case manager and I was fully employed for a week…So the reality is that case managers might be friends, patients themselves.

#### 3.3.2 Case conferences

Participants questioned why specialists continued to work in silos when structures (ie case conferences) already exist within cancer care for multidisciplinary team communication could be adapted to include and link with PCPs in the community, particularly where patients had other health and psychosocial care needs, where English was not their first language or where patients had other disabilities that impacted on their capacity to understand and cope.

#### 3.3.3 Shared decision making

Shared decision making was identified as needing to be embedded into shared care to support patients to make informed choices and decisions and to ensure that the patients' needs and preferences were clear to all concerned:

I think it's also valuable in the context that the expectation is that the patient will just hop on board to the model of care, but if you choose to go “No, hang on, I need to know what all my options are” or “Actually, I want to choose something different”, so that that's addressed right up front, and you're not sort of half way through the process of going “Oh actually I don't want to be on this track”, and having to get them off that track. So that's really valuable, so that everyone is on the same page and you've got those options addressed up‐front.

#### 3.3.4 Preparing patients for self‐management and cancer survivorship

When asked what aspects of shared care would be particularly worthy of pursuit, participants unanimously talked about the importance of prevention, early intervention, and preparing the patient for the journey of cancer survivorship.

I think it's also when someone is first diagnosed; it's actually preparing them – helping them to prepare. Early connection would be very desirable because then you can make choices. But then the better you are prepared, the better you are going to go through it too, so you gain a knowledge, so that's all shared.

#### 3.3.5 Building GPs' skills

Participants saw shared care commenced early as a valuable tool for building GPs' knowledge and skills and capacity to provide long‐term survivorship care. They described a process whereby GPs' familiarity, confidence, and skills could grow with shared care:

I suspect the same applies to the GP because they have an opportunity to be involved and engaged at the beginning, where they learn what happens as opposed to being kind of lumped with six months' worth of experience without being part of that experience…that's sort of – that's starting a little bit late.

#### 3.3.6 Measuring outcomes

Participants stressed the need for shared care to demonstrate its effectiveness; particularly its impact on patients' health outcomes, but also other measures of effectiveness such as cost benefits to the health system. They highlighted the assumption that shared care would be cost effective, though also discussed the need for more evidence to establish this.

For me, success would be…because it was shared that something was averted and it was like wow, because that was shared, it meant that that protected me…So shared care is not just a nice thing because you can, it's that you could say this is how it adds value for me. Yeah, it saved me.

## 4 DISCUSSION

The results of this examination of shared care for cancer survivors revealed a wealth of ideas about what is needed, how shared care can be advanced, what barriers need to be overcome, and what successful models of shared care might look like.

The results confirm that cancer survivors want to be at the centre of the process, to have active involvement, as part of a transparent person‐centred care approach, but that this is contrary to what happens for many cancer survivors who are frequently excluded from the planning of care, access to information, and shared decision making. Participants noted that there was a concerning gap between the rhetoric of person‐centred care and the reality of its delivery, given that all acknowledged cancer survivors and/or their family supports as the key navigators of care systems. They stressed the need for effective communication between health‐care providers across the various parts of health‐care delivery, and that this must include a mechanism that acknowledges the patient as the navigator.

Shared e‐health records and shared care planning were proffered as facilitators for improved communication and care coordination, though it was unclear who should lead the process within the current structure of care delivery. GPs were perceived as important to the coordination of long‐term survivorship care although they were also perceived to need more support to build their skills and confidence to take on that role. This concern has also been raised more broadly in the literature, despite the role of GPs as the primary contact and entry point into the health‐care system for most patients. A range of models of shared care exists within the chronic condition management space that could guide shared care in cancer.^12^ There is also emerging evidence for shared care models being acceptable to cancer survivors.^13^

Clear consensus and support exists for shared care that aligns with the recommendations of the Institute of Medicine (IOM) report.^14^ By this, they meant the need for greater emphasis on shared care, care plans, and care planning that involved all concerned, a point reiterated recently by Harris et al.,^15^ though these researchers also found that patients, GPs, and oncologists disagreed on what shared care should look like. This also involves effective care coordination to avoid duplication and improve communication and use of available human and service resources, and case conferencing to improve planning and on‐going provision of care. However, major concerns existed around the lack of good examples to help guide shared care for this population, and fragmentation of care and poor communication that continued to work against the implementation and embedding of shared care approaches into wider practice. These concerns are also apparent in chronic condition management, more broadly.^16^

All participants agreed that a self‐management focus should begin early in the cancer survivor journey and that recognizing cancer as a chronic condition requires a shift in how care is provided to these patients. However, they also expressed concern about the burden imposed on cancer survivors in navigating health systems that may not acknowledge or understand what self‐management might mean in this context. It is unclear how cancer survivors would respond to a greater emphasis on such an approach to shared care, due to the limited number of studies with this population available to guide us. What is known is that, in the presence of gaps in the health‐care system and the absence of effective coordination by services, people with chronic conditions (or their family carers) exercise a range of strategies (such as keeping personal medication lists) and must draw on or develop a level of personal agency (that is, a sense of personal control) to address these gaps.^17^ Cancer survivor participants in the current study provided numerous examples of their agency, in the absence of effective communication from health professionals and involvement in care.

This study also noted the importance of measures and data to determine efficacy of shared care for cancer survivorship. In the chronic condition management arena, a systematic review by Smith, Alllwright, and O'Dowd^18^ found limited quality evidence to demonstrate significant benefits of shared care arrangements. Similar findings were reported in a systematic review of integrated care studies.^19^ Smith et al.^18^ called for more research on the contextual conditions and mechanisms that influence how shared care works, arguing that, “Models of shared care can be viewed as classic complex interventions, being multi‐component, highly dependent on the behaviours and choices of those delivering and receiving the care, and having highly context‐dependent effectiveness. This creates challenges for conducting rigorous evaluations and also for synthesizing research evidence about shared care”(p.2). They argued, therefore, that a realist evaluation design would be valuable, to help explain, “how and why shared care and related models of care delivery are more or less effective in different circumstances or for different patient groups” (p.2). Cancer survivors represent one such population that may have unique circumstances. More recently, in the related area of self‐management support for chronic conditions, meta‐reviews and an implementation systematic review of self‐management support evidence^20^ concluded that it is inseparable from high‐quality care for people with chronic conditions and that enhanced communication between all involved was a pivotal component for success. A further systematic review of self‐management support^21^ found small improvements in quality of life, and some reduced utilization of health‐care, particularly in services support people with respiratory and cardiovascular conditions. The findings of these reviews may parallel the issues of importance for shared care; however, a more rigorous review of how this applies to cancer survivors is needed. Lyngsø et al.^22^ identified several instruments for measuring integrated care that might usefully guide such research, going forward.

This study has a number of important limitations. It involved a small sample of participants. Most participants were women, and most were advocates from various cancer survivor organizations. Though all had past or present experience with cancer, the views of the broader cancer population may not have been represented. Also, detailed collection of demographic information did not occur; hence, not all aged groups and cancer types and experiences may have been represented in the discussions. There were also a limited number of family caregivers present, and we recognize that their experience of system navigation and advocacy for the cared for person is likely important for shared care. The sample was therefore not sufficient to reach data saturation. We intend to undertake further work to canvass the views of specific cancer survivor population groups that may have been under‐represented, such as children, young people, non‐English speaking populations, and informal/family caregivers.

## 5 CONCLUSIONS

In conclusion, cancer survivors are generally supportive of the concept of shared care to deliver holistic care after cancer and identify a number of key elements that would ensure effectiveness of shared care after cancer, including effective communication, care coordination and navigation, and shared medical records. They acknowledge that such an approach requires preparation of both survivors and their health professionals and a framework of care that supports measurement of outcomes. Further research on how best to implement shared care model into the Australian setting that engages survivors in the process of planning and implementation is warranted.

## CONFLICTS OF INTEREST

The authors have no conflicts of interest to declare.
